# Untargeted Metabolomics Comparison and Nutrition Evaluation of Geographical Indication Newhall Navel Oranges in China

**DOI:** 10.3390/foods14030355

**Published:** 2025-01-22

**Authors:** Xiao Shu, Manli Xie, Xuemei Zhang, Na Wang, Wei Zhang, Junjie Lin, Junying Yang, Xiaoxia Yang, Yingkui Li

**Affiliations:** 1 Institute of Agricultural Quality Standard and Testing Technology, Chongqing Academy of Agricultural Sciences, Chongqing 401329, China; shuxiao1209@163.com (X.S.); mandju@163.com (M.X.); zhangxuemei-1@163.com (X.Z.); cqwangna@126.com (N.W.); zhangwei13-14@163.com (W.Z.); yjyqy5@163.com (J.Y.); 83761160@sohu.com (Y.L.); 2 School of Environment and Natural Resources, Zhejiang University of Science and Technology, Hangzhou 310023, China

**Keywords:** markers, amino acids, flavonoids, phenolic acids, terroir

## Abstract

The untargeted metabolomics of Newhall navel oranges from three areas in China—Ganzhou, Fengjie, and Zigui—with geographical indication (GI) was measured using LC-MS/MS. Orthogonal partial least squares discriminant analysis was performed for sample classification and important metabolite identification. This approach identified the best markers of the geographical origin able to discriminate Fengjie, Ganzhou, and Zigui orange samples. For peeled samples, 2-isopropylmalic acid, succinic acid, citric acid, L-aspartic acid, L-glutamic γ-semialdehyde, D-β-phenylalanine, hesperetin, hydrocinnamic acid, 4-hydroxycinnamic acid, and dehydroascorbate were the markers used to discriminate the geographical origin. All these markers were overexpressed in the peeled samples from the Zigui area, followed by the Ganzhou area. As for unpeeled samples, L-glutamic γ-semialdehyde, isovitexin 2′-O-β-D-glucoside, 2-isopropylmalic acid, isovitexin, diosmetin, trans-2-hydroxycinnamate and trans-cinnamate, L-aspartic acid, hydrocinnamic acid, and β-carotene were used to discriminate their origin. The first seven markers in Zigui-planted whole samples showed the highest levels, and the last three markers were richest in Ganzhou-planted samples. According to the variation in the markers for discriminating the origins of the peeled or unpeeled Newhall navel oranges with GI and the highest value of titratable acidity in those from Zigui, the samples planted in Ganzhou have the best balance between taste and nutrition. This work confirms that the approach of untargeted metabolomics combined with OPLS-DA is an effective way for origin tracing and overall quality evaluation.

## 1. Introduction

The geographical information of the planting locations for agricultural products has attracted more attention from consumers. For the same variety of fruit planted in different areas, even if cultivated using the same method, the quality may vary greatly, since the growing soil and climate can influence the quality of the fruit [1]. Therefore, authentic information on the geographical origin of agricultural products appears to be important in market transactions. To this end, the Ministry of Agriculture and Rural Affairs of China has proposed geographical indication (GI) for agricultural products, aiming to protect geographical origin brands and add potential economic value. Under this condition, there has been a phenomenon of counterfeit GI agricultural products in the market, which can affect the commercial values of GI agricultural products and mislead consumers. Thus, how to distinguish GI agricultural products effectively is one of the current research focuses.

Citrus, which is extensively cultivated in southern China, also holds the position of being the foremost in this region [2]. The Newhall navel orange (*Citrus sinensis* L. *Osbeck*) is a predominant citrus variety highly favored by Chinese consumers on account of its delectable taste, high moisture content, and potential nutrition value. The navel oranges grown in Ganzhou (GZ) City in the south of Jiangxi Province, Zigui (ZG) County in Hubei Province, and Fengjie (FJ) County in Chongqing City, have been incorporated into the list of GI agricultural products by the Ministry of Agriculture and Rural Affairs of the People’s Republic of China, signifying their superior quality. Nearly all GI navel oranges available in the market originate from these three areas. Present studies on citrus primarily concentrate on the impacts of diverse cultivation methods or varieties on conventional quality parameters (taste, flavor, etc.) [3] and routine chemical properties, such as soluble solids, titratable acids, vitamin C content, etc. [4]. In fact, assessing the chemical composition (metabolites) of economically important fruits, such as the Newhall navel orange, is of great theoretical value for the identification of the source of their origin and their scientific utilization. Although they all share the geographical indication as navel oranges, there may exist disparities in their chemical composition due to varying planting locations, which, in turn, gives rise to differences in their nutritional functions.

Without needing precise quantification using the standards, the combination of untargeted metabolomics and multivariate statistical orthogonal projection with latent structure discriminant analysis (OPLS-DA) has been shown as a potentially valuable tool for identifying the geographic origin and quality of agricultural products. Using this method, Ben Mohamed et al. [5] discovered that hydroxybenzoic acids, cholesterol, and stigmasterol derivatives are the optimal markers capable of discriminating the extra-virgin olive oil from Tunisia and Italia [6]. This method was also utilized to identify the differences between propolis from Greece and China, demonstrating that Chinese samples overexpressed compounds characteristic of the poplar type propolis, whereas Greek samples overexpressed those characteristic of the Mediterranean propolis type [7]. Thus, it is hypothesized that untargeted metabolomics in combination of OPLS-DA could identify the metabolite markers for discriminating the geographic origins of these three GI Newhall navel oranges as well. Additionally, the application of untargeted metabolomics enables the precise characterization of metabolite profiles, particularly the compounds correlated with flavor, such as sugars, acids, flavones, phenols, or other functional substances, disclosing the general differences in their flavor or nutrition [8,9,10,11].

The purpose of this study was (1) to explore the differences in the metabolic profiles of GI Newhall navel orange samples planted in the three different areas in China; (2) to verify whether untargeted metabolomics could identify the planting location of GI Newhall navel oranges; and (3) to further assess the nutrition of GI Newhall navel orange samples based on untargeted metabolomics. The results of this study will enhance the understanding of the characteristics of GI Newhall oranges planted in different areas and provide evidence for whether untargeted metabolomics can identify and distinguish the origins of geographical indication navel oranges.

## 2. Materials and Methods

### 2.1. Sampling

**Figure 1:**
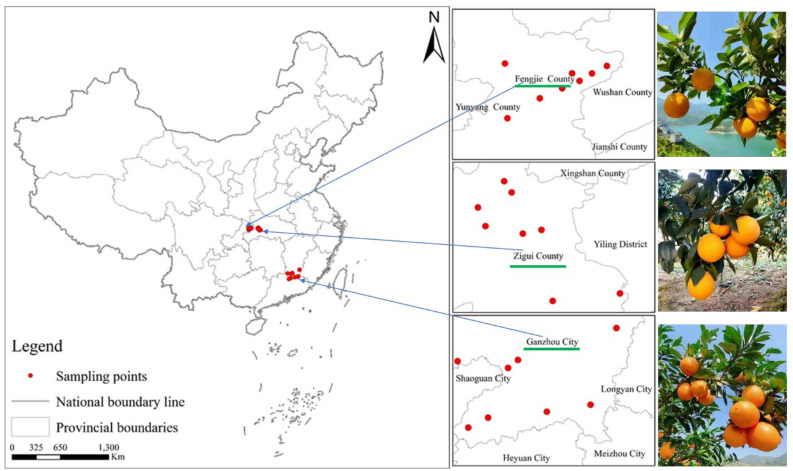
Locations of Newhall navel oranges with geographical indications from the areas Fengjie, Ganzhou, and Zigui in China.

Newhall navel oranges originated from three areas—Ganzhou, Fengjie, and Zigui—in China. Eight orchards were selectively picked from each of these areas. Because of the concentrated cultivation of navel oranges in the areas of Fengjie and Zigui, both of which are located in the Three Gorges Reservoir area of the Yangtze River in China and are geographically close to each other, the sampling orchards in these two places look almost overlapping on the map (Figure 1). The navel oranges underwent uniform field management and were harvested in December 2022 due to the slight variation in the ripening period from place to place. In total, 10 kg of oranges with a uniform size ranging from 65 to 70 mm in diameter were acquired.

**Figure 2:**
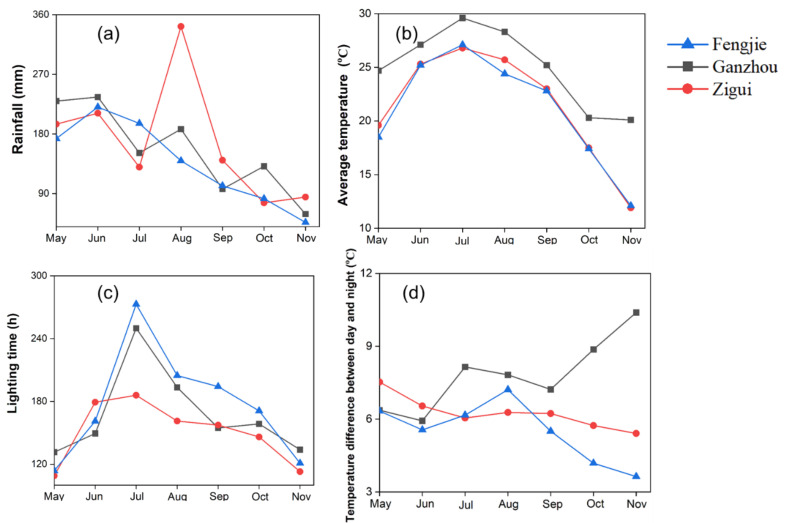
(**a**) the average monthly rainfall of Fengiie, Ganzhou, and Zigui from May to November, (**b**) the average monthly temperature of Fengiie, Ganzhou, and Zigui from May to November, (**c**) the average monthly lighting time of Fengiie, Ganzhou, and Zigui from May to November, (**d**) the average monthly temperature difference between day and night of Fengiie, Ganzhou, and Zigui from May to November.

The climatic data of the planting areas in 2022, including the rainfall, lighting time, average temperature, and temperature difference between day and night, obtained from the China Meteorological Administration (http://data.cma.cn, accessed on 5 June 2023), are shown in Figure 2. The maximum rainfall appeared in the Zigui area, and the average temperature in the Ganzhou region was higher than those of Fengjie and Zigui. The temperature difference between day and night in the three areas ranged from 3.4 °C to 10.6 °C. In the Ganzhou area, the temperature difference kept increasing from September to November and reached the maximum value in November, while it demonstrated the opposite trend in the other two areas. The amount of lighting in Fengjie and Ganzhou was similar, higher than that in Zigui.

The collected oranges were immediately stored in a chamber maintained at 4 °C with a relative humidity of 90% and subsequently transported to the laboratory. The soluble solids (SS), titratable acidity (TA), vitamin C (VC), and untargeted metabolomics were analyzed within three days.

### 2.2. Determination of Quality Attributes

The soluble solids (SS) content and titratable acidity (TA) of peeled or whole samples were determined in this work. The SS content was measured with a digital sugar refractometer (WAY-2S, Zenith Lab (Jiangsu) Co., Ltd, Changzhou, China), and TA was determined via sodium solution titration. Vitamin C (VC) was measured using the method of the 2, 6-dichloroindophenol (2, 6-D) solution titration. According to this method, the samples need to be peeled, meaning that only the VC data of peeled samples are available in the results.

### 2.3. Untargeted Metabolomics Analysis

#### 2.3.1. Sample Preparation

Four Newhall navel oranges were collected, and each was divided into four flaps. Subsequently, one-quarter were taken out and re-substituted as an unpeeled sample. To identify the differences in metabolic profiles resulting from peeling or not, another flap was peeled from each orange and reconstituted to a peeled sample. Thus, there were eight peeled samples and eight non-peeled samples from each terroir. The reconstituted samples designated for metabolite extraction were immediately frozen in liquid nitrogen, freeze-dried, grinded to obtain a homogeneous powder, and ultimately stored at −80 °C until extraction and analysis were carried out.

#### 2.3.2. Metabolite Extraction

Orange metabolites was extracted for LC-MS/MS analysis based on the method of Yang et al. [12]. The details were as follows: A total of 3 mL of iced methanol (80%, *v*/*v*) was added to 0.5 g of frozen orange powder. Then, the samples were placed in a high-throughput tissue grinder for 60 s. After sonicating for 30 min, the samples were transferred to ice for 20 min and centrifuged at 15,000× *g* for 20 min. Supernatants were concentrated during drying in a vacuum. Finally, the samples were reconstituted in acetonitrile (50%, *v*/*v*) and filtered through a 0.45 µm membrane for LC-MS/MS analysis.

#### 2.3.3. LC-MS/MS Analysis

The untargeted metabolomics were assessed using a Q-Extractive instrument interfaced with a heated electrospray ionization source and Orbitrap mass analyzer (Thermo Fisher Scientific, USA). The details of the LC-MS/MS parameters are referenced in a previous study by Yang et al. [12]. All samples were maintained at 4 °C, and gradient elution was performed with an Acquity UPLC HSS T3 (2.1 mm × 150 mm, 1.8 µm) (Waters, Milford, MA, USA) column at a flow rate of 0.25 mL/min. The mobile phase consisted of water (A) and acetonitrile (B), each containing 0.1% formic acid. The MS/MS analysis was executed using an electrospray ionization source, and data were acquired in both positive and negative modes. A primary full scan was conducted at 70,000 resolutions with a primary ion scan range of *m*/*z* 81–1000 and a secondary scan at 17,500 resolutions, while dynamic exclusion was employed to eliminate unnecessary MS/MS information.

#### 2.3.4. Data Processing

The LC-MS/MS data were converted into *m*/*z* XML format, and the metabolites were identified. Automatic peak detection, retention time calibration, and peak alignment were executed in XC-MS software (v.3.5.1). The batch effect was then eliminated by correcting the data based on QC samples. Metabolites with a relative standard deviation (RSD) > 30% in QC samples were filtered and then used for subsequent data analysis. The data matrix, including the mass-to-charge ratio, retention time, and peak area, was imported into SIMCA-P (V13, Umetrics, Umea, Sweden) for multivariate data analysis. Principal component analysis (PCA) was conducted to generate an overview of group clustering. OPLS-DA was conducted to obtain metabolites with significant intergroup differences to accentuate the differences between groups. The validity of the model was assessed using the model prediction rate Q^2^. Commonly, a relatively larger variable importance to projection (VIP) value indicates a relatively greater contribution to the classification model. Metabolites with ions with *p* < 0.01 and VIP > 1.0 were selected as important metabolites or named metabolite markers. For the markers with a VIP value ranking in the top 10, representing the top 10 most important metabolites for discriminating samples, the univariate patterns of the mass intensities were re-examined.

#### 2.3.5. Metabolite Identification

The process of metabolite identification for untargeted metabolomics was referred to in the general practice of previous studies [13,14,15] without using the standard solution for accurate quantification. However, prior to the experiment, standard solutions for each metabolite were analyzed using the same instrument and method to determine the retention times and characteristic ions used for the identification of peaks in unknown samples to build the metabolite database. Besides a self-built database, the metabolite databases Human Metabolome Database (http://www.hmdb.ca, accessed on 1 January 2024), massbank (http://www.massbank.jp/, accessed on 1 January 2024), LipidMaps (http://www.lipidmaps.org, accessed on 1 January 2024), mz cloud (https://www.mzcloud.org, accessed on 1 January 2024), KEGG (https://www.genome.jp/kegg/, accessed on 1 January 2024) were used for the retrieval and comparison of compounds. The molecular weight, adduct ions, and other information of metabolites were determined by the mass-to-charge ratio of parent ions in primary mass spectrometry, and the molecular formula was predicted. The predicted formula was subsequently compared and matched with the databases to achieve the primary qualitative identification of metabolites. At the same time, the metabolites containing secondary spectra in the quantitative list were compared and matched with the fragment ion information of each secondary spectrum in the above databases to achieve the secondary qualitative identification of metabolites.

### 2.4. Statistical Analyses

The error bars in figures represent the standard deviation (*n* = 8). Shapiro–Wilk and Levene’s tests were employed for verifying the normality and homogeneity of variance. Differences in indices between peeled or non-peeled samples in different areas were determined using analysis of variance (ANOVA) with Tukey’s post hoc test. Differences were significant at *p* < 0.01. Microsoft Excel, SPSS 16.0, and Origin 9.0 were applied for the data analysis and drawings.

## 3. Results

### 3.1. Soluble Solids Content, Titratable Acidity, and Vitamin C

**Figure 3:**
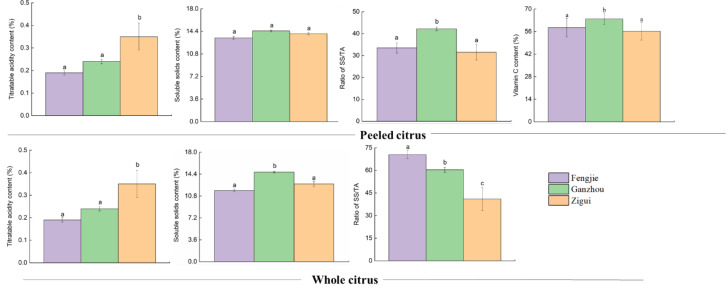
The titratable acidity, soluble solids, and vitamin C contents in peeled or whole citrus. The different lowercase letters above the error bars indicate the significant difference in the same index between samples with different origins under the same treatment.

As shown in Figure 3, the content of vitamin C in GI Newhall naval orange was in the order of Ganzhou > Fengjie > Zigui. In the whole or peeled samples, the titratable acidity was in the order of Zigui > Ganzhou > Fengjie, and the highest content of soluble solids were observed in Ganzhou, while the lowest was in Fengjie. The maximum SS/TA value was shown in peeled samples from Ganzhou. For the whole fruits, the maximum SS/TA value appeared in Fengjie-planted samples. Regardless of being peeled or not, the minimum SS/TA value occurred in Zigui-planted samples (Figure 3). It is worth noting that unlike SS or TA, vitamin C was not measured in the whole samples due to the requirement of peeling for determination.

### 3.2. Compound Identification

**Figure 4:**
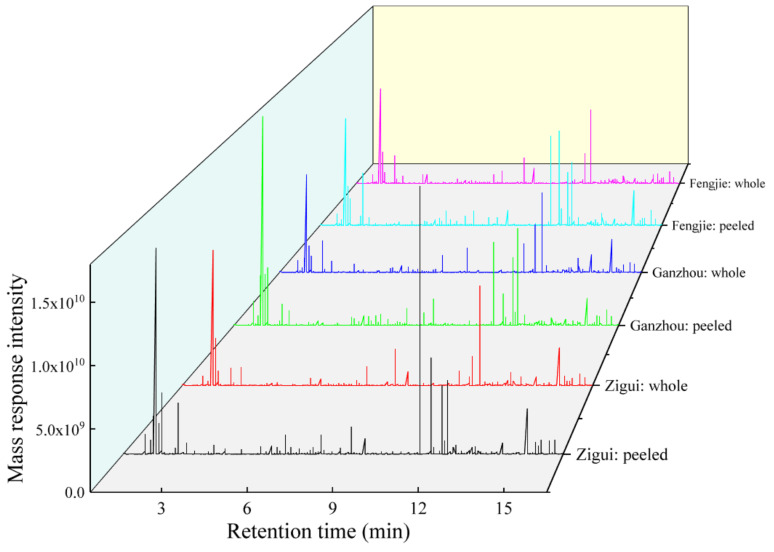
The total ion chromatogram of the peeled or whole citrus from Fengjie, Ganzhou, and Zigui in the positive mode.

The total ion chromatogram of the peeled or whole citrus from the Fengjie, Ganzhou, and Zigui areas in the positive mode (Figure 4) were presented, showing the summed intensity of all ions in the mass spectrum at different time points. Obvious variation can be observed on the chromatogram of the samples with different origins. It is worth mentioning that this work selected 2-chlorophenylalanine as the internal standard to assess the quality of the chromatographic data within each injection, since 2-chlorophenylalanine has good stability and solubility, a stable retention time, and easy electrospray ionization.

**Figure 5:**
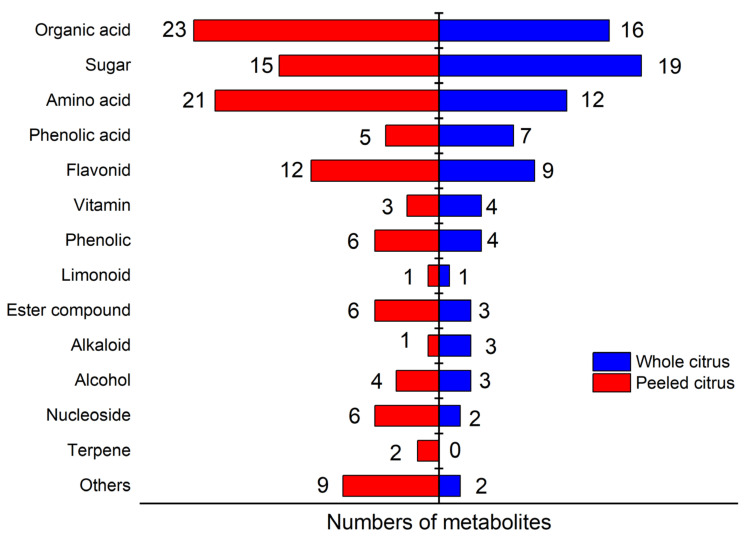
Classification and quantity of screened metabolites. “Others” refers to compounds that cannot be classified into the above categories.

To gain insight into the metabolites of the peeled or whole samples with different origins, we characterized these metabolic signals, including the retention time, exact molecular mass, characteristic fragments, etc. The identified compounds were classified into organic acids, amino acids, sugars, flavonoids, and so on (Figure 5). The identified information of the 114 metabolites detected in the peeled group and 85 metabolites in the whole group with VIP > 1 and *p* < 0.01 is presented in the Supplementary Materials Tables S1 and S2. For peeled samples, 23 organic acids were identified, 15 sugars, 21 amino acids, and 5 phenolic acids. In comparison, in the whole citrus, the number of identified compounds goes up to 16 for organic acids and 19 for sugars, declines to 12 for amino acids, and remains at 7 for phenolic acids.

### 3.3. Metabolic Marker Discrimination

#### 3.3.1. PCA, PLS-DA, and OPLS-DA Results

**Figure 6:**
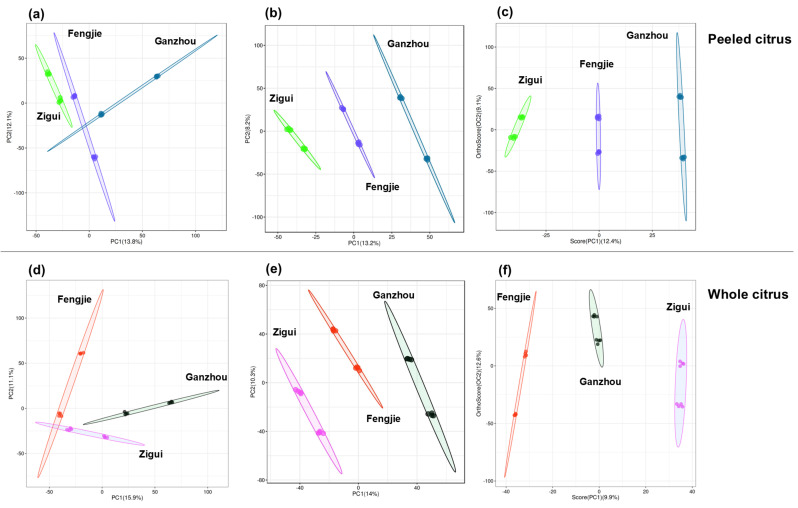
(**a**–**c**) are PCA, PLS-DA and OPLS-DA score plots of peeled citrus respectively. (**d**–**f**) are PCA, PLS-DA and OPLS-DA score plots of whole citrus respectively.

As illustrated in Figure 6a,d, the first two principal components (PCs) explained 25.9% and 27.0% of the total variance of the peeled and whole samples, respectively, and a poor separation between samples from Ganzhou and Fengjie was present. Partial least squares discriminant analysis (PLS-DA) is a supervised discriminant analysis that maximizes the differences between groups according to predefined classifications (Y variables), achieving better separation performance than PCA. Apparently, using PLS-DA, a better grouping of samples with different origins was shown in Figure 6b,e, respectively, and the prediction values of the model are both close to 0.96. As another multivariate statistical analysis, OPLS-DA offers superior model interpretability, a simplified model structure, and enhanced predictive performance compared to PLS-DA. Since OPLS-DA performs orthogonalization, the irrelevant components of *X* relative to *Y* could be eliminated, thus reducing the influence of noise on the model, minimizing redundant variables in the model, and improving its prediction accuracy [12]. Therefore, OPLS-DA has become a commonly used multivariate statistical method for analyzing metabolomics [16]. As illustrated in Figure 6c,f, a complete separation discrimination between the three classes of peeled or unpeeled samples was achieved from the OPLS-DA score plot as well. The model’s cross-validation parameters for the OPLS-DA were excellent, with *R*^2^*Y* and *Q*^2^*Y* having values of more than 0.98. The higher prediction level of OPLS-DA than that of PLS-DA suggests the obtained model from OPLS-DA for peeled or unpeeled samples is more reliable in comparison to PLS-DA. Therefore, we focused on the results obtained from OPLS-DA, especially the top 10 most important markers based on VIP values in the following analysis due to their greater contribution to the sample grouping and model prediction [12].

#### 3.3.2. Top Ten Most Important Markers

**Figure 7:**
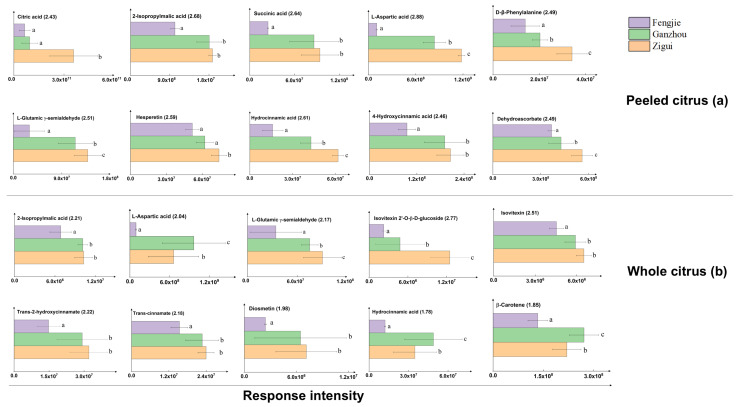
The response intensity of the top 10 most important metabolites identified using untargeted metabolomics in the peeled (**a**) and whole citrus (**b**) samples on the mass spectrum. The number in parentheses in each subgraph represents the variable importance to projection value of this metabolite. Different lowercase letters indicate significant difference between areas (*p* < 0.01).

With regard to peeled citrus, the top 10 most important markers were categorized into five groups: (1) the carboxylic acids 2-isopropylmalic acid, succinic acid, and citric acid, with VIP values of 2.68, 2.64, and 2.43, respectively; (2) the amino acids L-aspartic acid, L-glutamic γ-semialdehyde, and D-β-phenylalanine, with VIP values of 2.88, 2.51, and 2.49, respectively; (3) phenolic compounds, including the flavonoid hesperetin, with a VIP value of 2.59; (4) two phenolic acids, hydrocinnamic acid and 4-hydroxycinnamic acid, with VIP values of 2.61 and 2.46, respectively, and (5) active substances, like dehydroascorbate (DHA), with VIP value 2.49, which cannot be strictly classified as sugars, organic acids, or flavonoids (Figure 7a).

The level of citric acid was about three orders of magnitude higher than that of succinic acid (Figure 7a). The content of 2-isopropylmalic acid is approximately one order of magnitude lower than that of succinic acid (Figure 7a). Among GI navel oranges from the three terroirs, the citric acid in peeled navel oranges planted in Zigui was about three times higher than that in samples planted in the other two areas, representing the high acidity of the Zigui sample. Compared to the peeled samples planted in Zigui area, the contents of 2-isopropylmalic acid and succinic acid in Ganzhou-planted peeled samples were slightly higher but not significantly. Since the level of citric acid determines the acidity and even taste of the sample, our results indicate that Zigui-planted peeled samples possess the highest acidity. Since L-aspartic acid had the highest VIP value among the top 10 most important markers, it could be regarded as the most important metabolite marker. Three amino acid markers were similarly distributed among the samples from the three terroirs, with the highest levels appearing in the samples from Zigui and the lowest values in the samples from Fengjie (Figure 7a). Similarly, the amino acids, the highest level of hesperetin, the phenolic acid hydrocinnamic acid, and 4-hydroxycinnamic acid markers or DHA were found in peeled samples planted in Zigui, followed by Ganzhou and Fengjie (Figure 7a).

As for the whole samples, among the top ten most important markers, two amino acid markers—L-aspartic acid and L-glutamic γ-semialdehyde—were observed, with corresponding VIP values of 2.04 and 2.17, and both were found as the most important metabolites in peeled samples as well (Figure 7b). The number of carboxylic acid markers decreased to one, namely 2-isopropylmalic acid, with the VIP value 2.21. The number of phenolics increased to six, including isovitexin 2′-O-β-D-glucoside, isovitexin, diosmetin, trans-2-hydroxycinnamate, trans-cinnamate, and hydrocinnamic acid, the last of which is observed as the marker in the peeled samples as well. The VIP values of isovitexin 2′-O-β-D-glucoside and isovitexin are 2.77 and 2.51, respectively, ranking as the first and second, showing that they made a significant contribution in distinguishing peeled citrus samples. Unlike peeled samples, the active substance-like marker in unpeeled samples was β-carotene, with the VIP value 1.85 (Figure 7b).

All the top ten most important markers in whole citrus from the Fengjie area reached their lowest level (Figure 7b). The distribution of the compounds 2-isopropylmalic acid, isovitexin, trans-2-hydroxycinnamate, trans-cinnamate, and diosmetin in whole citrus originating from different areas is similar. That is, all of them were slightly more abundant in the Zigui-planted samples than in the Ganzhou-planted samples. Different from the variation in markers in peeled samples, L-aspartic acid, hydrocinnamic acid, and β-carotene were all significantly higher in unpeeled citrus samples from Ganzhou than those from Zigui. On the contrary, L-glutamic γ-semialdehyde and isovitexin 2′-O-β-D-glucoside were found to be significantly higher in samples from Zigui than in samples with peels from Ganzhou (Figure 7b).

### 3.4. Nutrition Evaluation

The sugar–acid ratio generally determines the taste of the fruit [1]. In both peeled and unpeeled samples, the sugar–acid ratio was in descending order, as Fengjie > Ganzhou > Zigui. This indicates that the taste is the best from Fengjie and the worst from Zigui.

After removing the organic acids from the top 10 most important markers, we further calculated the total nutritional score of the samples (with or without peel), and the results are presented in Table 1. The details about the calculation of the nutritional score are presented as follows: For a certain compound, the lowest significant level was assigned as 1. If no significant difference from the lowest level was observed, a value of 1 was still assigned. A value of 2 was assigned if there was no significant difference from the highest level, and the highest significant level was assigned as 3. After assigning metabolic markers one by one, the final total nutritional value of the samples (with or without peel) planted in a certain area is obtained by adding them up.

As demonstrated in Table 1, the highest nutritional score for peeled samples was found in Zigui, followed by Ganzhou and Fengjie. Differently, the whole samples possessed slightly higher nutritional score values for Ganzhou than those from Zigui, and the lowest score was found in the whole samples from Fengjie. Thus, the samples in Zigui have higher nutritional value and the worst taste due to having the lowest sugar–acid ratio. The samples from Ganzhou achieved the best balance between nutrition and taste.

## 4. Discussion

### 4.1. Quality Attributes

VC in fruits is one of the most important health-promoting traits [14]. The soluble solids content can reflect internal quality characteristics for the taste of fruits and affect the consumer acceptance of products [15]. A high content of VC represents high nutrition, and a high level of SS indicates a good taste [14]. The samples from Ganzhou possessed the highest VC and SS contents (Figure 3), suggesting that the samples from Ganzhou have the best taste and highest nutrition. The higher TA means a more acidic taste [14]. Regardless of the presence of peels, the Zigui sample is the most acidic with the maximum TA value. Since the higher SS/TA ratio represents the higher maturity level and better flavor [15], the Zigui samples, having the minimum value of the SS/TA ratio, appeared to have the lowest ripeness and worst flavor (Figure 3). Thus, the best taste and highest nutrition occurred in Ganzhou-planted samples, as determined through the VC, SS, and TA analysis.

### 4.2. The Discrimination of GI Orange Samples and Top Ten Most Important Markers

Multivariate analysis of the untargeted metabolomics is usually performed by applying supervised tools, such as PLS-DA and OPLS-DA, together with unsupervised methods, such as PCA. Since OPLS-DA could effectively separate Y-predictive variation from Y-uncorrelated variation in X, an unparalleled advantage in terms of sample grouping was demonstrated. Without using a standard solution for precise quantification, the metabolite markers can be screened based on the response intensity of the mass using untargeted metabolomics combined with OPLS-DA, and these markers are able to authenticate the origin of agricultural products [17,18].

In our work, from the OPLS-DA score plots, a complete separation between peeled or whole samples with different geographical origins was presented compared to PCA (Figure 6). In addition, the values of R^2^Y and Q^2^Y of more than 0.9 suggest the robustness of OPLS-DA to discriminate GI orange samples according to geographic origin. These preliminary results suggest that untargeted metabolomics analysis followed by the robust OPLS-DA model could be efficient approach for studying GI orange traceability. Subsequently, the variables’ importance to projection (VIP) in the OPLS-DA model was analyzed, considering the VIP score for each compound identified. Commonly, the variables with VIP > 1.0 could be considered to mostly contribute to class grouping in the OPLS-DA model. In current work, the number of the markers with a VIP value greater than 1.0 exceeded fifty. Since a higher VIP value of the markers possessed represents a greater contribution to class discrimination, the markers possessing the top ten highest VIP values in the supervised OPLS-DA model received particular attention. For peeled or whole samples, the top ten most important markers differed significantly, but these best markers were mainly attributed to carboxylic acids, amino acids, and phenolic compounds.

For peeled samples, three carboxylic acids (citric acid, succinic acid, and 2-isopropylmalic acid) were identified as the top ten important markers. Citric acid is well known as the predominant organic acid in citrus [19,20]. We found that the level of citric acid is three orders and four orders of magnitude higher than that of succinic acid and 2-isopropylmalic acid in peeled samples (Figure 7a), respectively. The above three organic acids reached the maximum level in the peeled-samples from Zigui (Figure 7a), indicating that the TCA cycle was more active in Zigui-planted samples than those from the other two areas. Previous studies discovered that the generation of tricarboxylic acids depended on the environmental condition, especially the temperature, and that a rising temperature could accelerate the metabolic rate of fruits, hence reducing the content of tricarboxylic acids [1,21]. We agree with the above inference that the temperature in Zigui area is indeed lower than that in Ganzhou area (Figure 2), and the lower temperature leads to the accumulation of tricarboxylic acids [22]. No sugars appeared in the top 10 most important markers, reflecting that the levels of sugars in GI navel oranges with different geographical origins are comparable, with no significance. Due to the highest levels of organic acids in the samples from the Zigui area, obviously, the value of the sugar–acid ratio, which reflects the citrus flavor, is the smallest in the samples from the Zigui area. As the fruit ripens, the organic acid level decreases simultaneously, and the maturity of fruit can, thus, be judged by the acidic or sweet flavors [23]. The highest level of acids occurring in the Zigui-planted peeled samples suggests that the maturity of samples from this area is lower than those from the other two areas, which is coincident with the results of SS/TA (Figure 3).

Amino acids participate in fruit quality formation together with other flavor substances. Also, fruits with higher contents of amino acids are more nutritious [24]. Both L-aspartic acid and L-glutamic γ-semialdehyde were observed to be among the top ten important markers contributing to discriminating the samples (peeled or unpeeled) with diverse origins (Figure 7). The variations in these amino acids in peeled samples directly mirror their flavor disparities [1]. The intermediate products of the TCA cycle can be converted into amino acids, among which citric acid can be directly converted into glutamic acid through the GABA pathway, and 2-ketoglutaric acid is involved in the formation of L-glutamic γ-semialdehyde [25]. This can help us understand why the distribution of amino acid markers in Newhall navel oranges grown in different places is similar with that of organic acids. Similar to our finding, the previous studies by Zhu [26] and Behrooz Nateghpour et al. [27] found that aspartic acid was the main chemical component of the amino acids in citrus pulp and peel. Consistently, amino acids (such as L-aspartic acid) and carboxylic acids (such as citric acid and succinic acid) were also reported to be markers that distinguish citrus fruit juice harvested in the state of Veracruz, Mexico [28]. However, the details on how amino acids and citric acid correlate exactly are still unknown.

Phenolic compounds, including flavonoids and phenolic acid, are associated with a variety of health benefits [29], such as natural antioxidant, anti-inflammatory, immunomodulatory, and anti-cancer activities, and also contribute to the flavor quality of navel oranges [30,31]. Flavonoid and phenolic acids usually reflect typical sensory properties such as color, astringency, and irritability as well [32,33]. The flavonoid hesperetin and the phenolic acids hydrocinnamic acid and 4-hydroxycinnamic acid were observed in the top 10 markers to distinguish peeled samples with different geographical origins (Figure 7b). The formation of phenolic acids is closely related to the amino acid metabolism [34,35]. Based on KEGG analysis, the generation of hydrocinnamic acid and trans-2-hydroxycinnamate can be affected by phenylalanine metabolism. Higher phenylalanine naturally produces more phenolic acids [36]. The difference in phenylalanine levels among the peeled samples can explain the phenomenon that the peeled samples in Zigui had the highest level of hydrocinnamic acid and 4-hydroxycinnamic acid, followed by Ganzhou and Fengjie (Figure 7a). In line with our results, Chen et al. [37] also found that hesperetin was the most abundant flavanone in Chinese citrus and the richest in Zigui peeled citrus. Different from our results, Li et al. [1] found that hesperetin in navel oranges from the Zigui and Fengjie areas was more prominent than those from Ganzhou area. They concluded that this could be attributed to the lower temperature in the Zigui area compared to the Ganzhou area, which has been reported to contribute to the upregulation of flavonoid biosynthesis-related genes at low temperatures, thereby increasing the production of flavonoids [38]. We agree with the above explanation. However, we observed the significance in the hesperetin content between samples from the Zigui and Fengjie areas, although the climates of these two areas are very similar, including their temperatures (Figure 2). This difference indicates that besides temperature, there are other factors that can affect the metabolic profile of Newhall navel oranges.

Diosmetin can inhibit carcinogenic activation and fight against lipopolysaccharide-induced acute lung injury by inhibiting nucleotide-binding domain-like receptor protein 3 inflammasome and activating nuclear factor erythroid 2–related factor 2 [39]. The contents of hesperidin and diosmetin in fruits can increase greatly at 90–120 days after flowering and then begin to decline [40]. Thus, the maturity of samples should be lower from the Zigui area than others due to the higher levels of diosmetin and hesperidin (Figure 7b), which is in line with the results of SS/TA (Figure 3). From the perspective of the nutrients of the fruit peel, navel oranges originated from Ganzhou or Zigui should be preferable. In many citrus fruits, VC is an inherent component that is naturally dissolved in the hydrophilic fraction. DHA is the reversible oxidizing form of VC. Since the ratio of reduction to oxidation forms of VC is approximately 15:1, the reduction form of VC is used to reflect the level of VC [41]. We observed that in peeled samples, VC is most abundant in those from Ganzhou, followed by Zigui, and DHA is the highest in Zigui, followed by Ganzhou. Although the DHA level is minimal compared to reduction VC, it does have its unique health benefits. Clinical trials found that pre-treatment with DHA can protect the brain in a shocked mouse, increasing blood flow after ischemia, and reducing nerve damage before reversible occlusion occurs, while VC does not have this effect [41]. By combining the results of DHA with VC in peeled samples, the nutritional value of the Ganzhou and Zigui samples seems to be higher than that of the Fengjie samples. β-carotene, as a natural vitamin, has potential health benefits in preventing chronic diseases, including age-related macular degeneration and cataracts and cardiovascular disease [42,43]. The β-carotene level was much higher in Ganzhou-peeled samples followed by Zigui samples (Figure 7a), indicating that navel oranges produced in the Ganzhou area may have better health-promoting properties compared to those produced in the areas of Fengjie and Zigui. Similar results were demonstrated in the study by Villa-Ruano et al. [31], who found that VC can be used as a differential metabolite to distinguish juice made from different citrus fruits. Based on their special nutritional properties, Newhall navel oranges produced in the Ganzhou or Zigui areas are in higher demand than those produced in the Fengjie area. This demand may be partly due to higher levels of VC and β-carotene.

Differently from the top ten most important markers screened from the peeled sample, in whole citrus, the number of organic acid metabolic biomarkers decreased, and the number of phenolic compounds increased. This phenomenon is mainly attributed to the presence of the peel, which account for 20–35% of the fruit weight and possesses rich essential oils, flavonoids, and pigments. Our results suggest that the peel should be a valuable food resource. Being consistent with our results, some previous studies found that in citrus fruits, phenolic compounds are more abundant in the skin than in the flesh [37,43,44]. Therefore, if the nutritional benefits of flavonoids are to be fully utilized, it is crucial to increase the utilization of the peel [40,45].

In summary, in this work the identification of markers including carboxylic acids, amino acids and phenols using untargeted metabolomics followed by multivariate statistics could be exploited to discriminate samples according to their geographical origin. Using a similar method to us, Ben et al. [5] identified phenolic and steroid markers in Tunisian and Italian extra-virgin olive oil samples. Kasiotis et al. [46] disclosed the potential markers as flavonoids, terpenoids, iridoids, and fatty acid ester derivatives for the differentiation of the three orange blossom honeys from Italy, Greece, and Egypt, respectively. The combined use of carboxylic acids, amino acids, and phenols is a promising method, likely since these three classes have different biosynthetic pathways, which have been discussed in this section, and thus might be differentially affected by pedo-climatic factors (the details can be seen in Section 4.4). In addition, these above markers can also indicate the variation in the quality or the nutrition of the samples according to their geographical origins. Nevertheless, experiments around the entire growth cycle of the species are further needed to understand the source of the variation.

### 4.3. Nutritional Comparison

With the exception of acids, important markers (including amino acids, phenols, and other active substances), screened using untargeted metabolomics, have been reported as nutrients [47,48]. In fact, evaluating the nutrition of foods is complex and still challenging. The Nutrient-Rich Food (NRF) model, an internationally recognized method for evaluating food nutrition, is an integrated multiple-index method based on nutrient density [47], which is the ratio of the nutrient composition of a food to the nutrient requirements of the human consumer. However, the corresponding nutrient density for most of the important markers in this study is still unknown [49]. To compare the nutritional value of the samples, we attempted to apply the integrated index based on the statistical results.

As shown in Table 1, there are close scores for the nutritional evaluation between the whole samples from the areas of Ganzhou and Zigui, with these being much higher than that of the Fengjie area, and the final nutrition score is ordered from Ganzhou > Zigui > Fengjie. For peeled samples, the final nutrition score is ordered from Zigui > Ganzhou > Fengjie, suggesting that the presence of the peel has a prominent effect on the nutrition of oranges. However, the taste of the samples from the Zigui area is the worst, as evidenced by the lowest sugar–acid ratio. Therefore, navel oranges cultured in the Ganzhou area are more palatable in taste and flavor and higher in nutritional value. Our conclusion coincides with that of previous study by Li et al. [1], who found that peeled Newhall navel oranges produced in the Zigui area have higher nutrition, as evidenced by the higher levels of flavonoids and tricarboxylic acid, than in those produced in other two areas. It seems that the untargeted metabolomics results are not completely consistent with the conclusions obtained solely based on VC, which supports the necessity of combining untargeted metabolomics approaches in this study. Because the untargeted metabolomics results cover more compounds, the conclusions based on untargeted metabolomics are preferred. Actually, some scholars have also reached similar conclusions that the nutritional value of fruits could be evaluated using their metabolite profiles rather than the routine conventional parameters (such as VC) [14]. It is worth noting that no perfect food nutrition evaluation method has been reported till now, and the nutrition evaluation method applied in this study can provide some insights into the nutrition of Newhall navel oranges.

### 4.4. The Potential Association Between the Terroir and the Quality of Navel Orange

Climatic factors, such as temperature, rainfall, and the temperature difference between day and night, may affect the quality of fruits as well [14]. Higher temperatures and larger temperature differences between the day and night favor photosynthesis and sugar accumulation in fruits, resulting in higher fruit sweetness. Among the three areas studied, both the average temperature and the difference in the temperature between day and night in the Ganzhou area are higher than those in the remaining two areas (Figure 2), which helps to explain our observation that Ganzhou-planted navel oranges have a good taste and low acidity. The expansion period of navel orange fruit usually occurs in August or September, and excessive rainfall is unfavorable for the accumulation of sugar. The rainfall in the Zigui area during this period was apparently higher than in the other two planting areas (Figure 2), and this may contribute to the highest level in organic acid metabolites in the samples.

The soil pH can directly influence the availability of nutrients for plant growth and further affect the quality of fruits. The previous study reported that a soil pH ranging from 4.8 to 5.5 in the acidic range and from 6.6 to 7.8 in the alkaline range are suitable for the growth of Newhall navel oranges [1]. Most of the soil in the Ganzhou Newhall navel orange planting area was red soil with a pH about 4.8, and in the areas of Zigui and Fengjie, the soil pH was between 7.0 and 7.1 [1,50]. Differences in the soil pH may contribute to differences in metabolic markers in samples across areas. These findings indicated that the differences in the untargeted metabolomics in oranges from different areas were the result of a combination of many factors, including the climate, soil, etc. Although the average temperature and soil acidity in the areas of Zigui and Fengjie are similar, the GI navel oranges planted in both two areas have significant differences in their taste and nutrition, indicating that there are other factors existing that affect the growth of navel oranges. However, the details are still unknown and need further research.

## 5. Conclusions

2-isopropylmalic acid, succinic acid, citric acid, L-aspartic acid, L-glutamic γ-semialdehyde, D-β-phenylalanine, hesperetin, hydrocinnamic acid, 4-hydroxycinnamic acid, and dehydroascorbate were the markers used to discriminate the geographical origin of peeled citrus with GI. As for unpeeled samples, L-glutamic γ-semialdehyde, isovitexin 2′-O-β-D-glucoside, 2-isopropylmalic acid, isovitexin, diosmetin, trans-2-hydroxycinnamate and trans-cinnamate, L-aspartic acid, hydrocinnamic acid, and β-carotene were able to discriminate the geographical origin. Their variation should be closely related to the superiority of climate and soil conditions. We believe that the obtained metabolite profiles of citrus with GI but different origins will enhance the knowledge on the quality of navel oranges and contribute to further improving and scientifically utilizing the quality of navel oranges.

## Figures and Tables

**Table 1:** Nutritional assessment of the peeled and whole citrus samples from Fengjie (FJ), Ganzhou (GZ), and Zigui (ZG) based on the ten most important metabolites selected from peeled or peeled samples after removing the organic acids, respectively.

Samples	Number	Metabolites	Assigned Score			Mass Error (ppm)
			FJ	GZ	ZG	
Peeled	1	L-aspartic acid	1	2	3	0.68
	2	D-β-phenylalanine	1	2	3	9.90
	3	L-glutamic γ-semialdehyde	1	2	3	16.77
	4	Hesperetin	1	1	2	2.28
	5	Hydrocinnamic acid	1	2	3	29.56
	6	4-hydroxycinnamic acid	1	2	2	4.69
	7	DHA	1	2	3	6.52
		Total score	7	13	19	
Whole	1	L-aspartic acid	1	3	2	0.68
	2	L-glutamic-semialdehyde	1	2	3	16.77
	3	Isovitexin 2′-O-β-D-glucoside	1	2	3	0.80
	4	Isovitexin	1	2	2	4.56
	5	Trans-2-hydrocinnamate	1	2	2	2.28
	6	Trans-cinnamate	1	2	2	2.40
	7	Diosmetin	1	2	2	1.08
	8	Hydrocinnamic acid	1	3	2	29.56
	9	β-carotene	1	3	2	0.85
		Total score	9	21	20	

## Data Availability

The original contributions presented in this study are included in the article. Further inquiries can be directed to the corresponding author.

## Supplementary Materials

The following supporting information can be downloaded at https://www.mdpi.com/article/10.3390/foods14030355/s1: Table S1: The details of 114 detected compounds with VIP > 1 and *p* < 0.01 in the peeled citrus group; Table S2: The details of 85 detected compounds with VIP > 1 and *p* < 0.01 in the whole citrus group.

## Abbreviations

GI, geographical indication; LC-MS/MS, liquid chromatography–tandem mass spectrometry; PLS-DA, partial least squares discriminant analysis; OPLS-DA, prthogonal partial least squares discriminant analysis; GZ, Ganzhou; ZG, Zigui; FJ, Fengjie; SS, soluble solids; TA, titratable acidity; VC, vitamin C; PCA, principal component analysis; VIP, variable importance to projection; PCs, principal components; ANOVA, analysis of variance; DHA, dehydroascorbate; TCA, tricarboxylic acid; GABA, gamma-aminobutyric acid; KEGG, Kyoto Encyclopedia of Genes and Genomes; NRF, Nutrient-Rich Food.

## References

[1] Li Y., Liang L., Xu C., Yang T., Wang Y. (2021). UPLC-Q-TOF/MS-based untargeted metabolomics for discrimination of navel oranges from different geographical origins of China. LWT.

[2] Ollitrault P., Terol J., Chen C., Federici C.T., Lotfy S., Hippolyte I., Ollitrault F., Berard A., Chauveau A., Cuenca J. (2012). A reference genetic map of *C. clementina* hort. ex Tan.; citrus evolution inferences from comparative mapping. BMC Genom..

[3] Lin H., He C., Liu H., Shen G., Xia F., Feng J. (2021). NMR-based quantitative component analysis and geographical origin identification of China’s sweet orange. Food Control.

[4] Hu D.-Y., Liao Q.-H., Xie R.-J., He S.-L., Qian C., Lv Q., Yi S.-L., Zheng Y.-Q., Deng L. (2015). Effect of Geographical Location on Physical Characteristics and Chemical Compositions of Newhall Navel Orange (*Citrus sinensis* (L.) Osbeck). Food Sci..

[5] Ben Mohamed M., Rocchetti G., Montesano D., Ben Ali S., Guasmi F., Grati-Kamoun N., Lucini L. (2018). Discrimination of Tunisian and Italian extra-virgin olive oils according to their phenolic and sterolic fingerprints. Food Res. Int..

[6] Sales C., Cervera M.I., Gil R., Portolés T., Pitarch E., Beltran J. (2017). Quality classification of Spanish olive oils by untargeted gas chromatography coupled to hybrid quadrupole-time of flight mass spectrometry with atmospheric pressure chemical ionization and metabolomics-based statistical approach. Food Chem..

[7] Stavropoulou M.I., Termentzi A., Kasiotis K.M., Cheilari A., Stathopoulou K., Machera K., Aligiannis N. (2021). Untargeted ultrahigh-performance liquid chromatography-hybrid quadrupole-orbitrap mass spectrometry (UHPLC-HRMS) metabolomics reveals propolis markers of Greek and Chinese origin. Molecules.

[8] Colantonio V., Ferra L.F.V., Tieman D.M., Bliznyuk N., Sims C., Klee H.J., Munoz P., Resende M.F.R. (2022). Metabolomic selection for enhanced fruit flavor. Proc. Natl. Acad. Sci. USA.

[9] Septembre-Malaterre A., Remize F., Poucheret P. (2018). Fruits and vegetables, as a source of nutritional compounds and phytochemicals: Changes in bioactive compounds during lactic fermentation. Food Res. Int..

[10] Wang X.L., Wu L.X., Qiu J., Qian Y.Z., Wang M. (2023). Comparative metabolomic analysis of the nutritional aspects from ten cultivars of the strawberry fruit. Foods.

[11] Pu J., Vinitchaikul P., Gu Z., Mao H., Zhang F. (2021). The use of metabolomics to reveal differences in functional substances of milk whey of dairy buffaloes raised at different altitudes. Food Funct..

[12] Yang X.X., Gong J.P., Zhang X.M., Huang Y.C., Zhang W., Yang J.Y., Lin J.J., Chai Y., Liu J.F. (2021). Evaluation of the combined toxicity of multi-walled carbon nanotubes and cadmium on earthworms in soil using multi-level biomarkers. Ecotoxicol. Environ. Saf..

[13] Liu Y., Liu J., Liu M., Liu Y., Strappe P., Sun H., Zhou Z. (2020). Comparative non-targeted metabolomic analysis reveals insights into the mechanism of rice yellowing. Food Chem..

[14] Xu L., Wang L., Xu Z., Zhang X., Zhang Z., Qian Y. (2021). Physicochemical quality and metabolomics comparison of the green food apple and conventional apple in China. Food Res. Int..

[15] Li Q., Yang S., Li B., Zhang C., Li Y., Li J. (2022). Exploring critical metabolites of honey peach (*Prunus persica* (L.) Batsch) from five main cultivation regions in the north of China by UPLC-Q-TOF/MS combined with chemometrics and modeling. Food Res. Int..

[16] Wang Z., Chen X., Liu Q., Zhang L., Liu S., Su Y., Ren Y., Yuan C. (2023). Untargeted metabolomics analysis based on LC-IM-QTOF-MS for discriminating geographical origin and vintage of Chinese red wine. Food Res. Int..

[17] Pimenta J.V.C., dos Santos L.B., Almeida M.R., Augusti R., de Macedo A.N. (2025). Geographic origin characterization of Brazilian green coffee beans via untargeted metabolomics. Food Chem..

[18] He L., Hu Q., Zhang J., Xing R., Zhao Y., Yu N., Chen Y. (2023). An integrated untargeted metabolomic approach reveals the quality characteristics of black soybeans from different geographical origins in China. Food Res. Int..

[19] Huang Y., He J., Xu Y., Zheng W., Wang S., Chen P., Zeng B., Yang S., Jiang X., Liu Z. (2023). Pangenome analysis provides insight into the evolution of the orange subfamily and a key gene for citric acid accumulation in citrus fruits. Nat. Genet..

[20] Katz E., Boo K.F., Kim H.Y., Eigenheer R.A., Phinney B.S., Shulaev V., Negre-Zakharov F., Sadka A., Blumwald E. (2011). Label-free shotgun proteomics and metabolite analysis reveal a significant metabolic shift during citrus fruit development. J. Exp. Bot..

[21] López-Bucio J., Nieto-Jacobo M.F., Ramírez-Rodríguez V., Herrera-Estrella L. (2000). Organic acid metabolism in plants: From adaptive physiology to transgenic varieties for cultivation in extreme soils. Plant Sci..

[22] Kim H.J. (2021). Comparative Metabolomics Analysis of Citrus Varieties. Foods.

[23] Jayarajan S., Sharma R.R., Sethi S., Saha S., Sharma V.K., Singh S. (2019). Chemical and nutritional evaluation of major genotypes of nectarine (*Prunus persica* var *nectarina*) grown in North-Western Himalayas. J. Food Sci. Technol..

[24] Sogvar O.B., Rabiei V., Razavi F., Gohari G. (2020). Phenylalanine alleviates postharvest chilling injury of plum fruit by modulating antioxidant system and enhancing the accumulation of phenolic compound. Food Technol. Biotechnol..

[25] Cercos M., Soler G., Iglesias D.J., Gadea J., Forment J., Talon M. (2006). Global analysis of gene expression during development and ripening of citrus fruit flesh. A proposed mechanism for citric acid utilization. Plant Mol. Biol..

[26] Zhu C., Lu Q., Zhou X., Li J., Yue J., Wang Z., Pan S. (2020). Metabolic variations of organic acids, amino acids, fatty acids and aroma compounds in the pulp of different pummelo varieties. LWT.

[27] Nateghpour B., Kavoosi G., Mirakhorli N. (2021). Amino acid profile of the peel of three citrus species and its effect on the combination of amino acids and fatty acids Chlorella vulgaris. J. Food Compos. Anal..

[28] Villa-Ruano N., Pérez-Hernández N., Zepeda-Vallejo L.G., Quiroz-Acosta T., Mendieta-Moctezuma A., Montoya-García C., García-Nava M.L., Becerra-Martínez E. (2019). ^1^H-NMR based metabolomics profiling of citrus juices produced in Veracruz, México. Chem. Biodivers..

[29] Rashmi H.B., Negi P.S. (2020). Phenolic acids from vegetables: A review on processing stability and health benefits. Food Res.Int..

[30] Anantharaju P.G., Gowda P.C., Vimalambike M.G., Madhunapantula S.V. (2016). An overview on the role of dietary phenolics for the treatment of cancers. Nutr. J..

[31] Stuper-Szablewska K., Perkowski J. (2017). Phenolic acids in cereal grain: Occurrence, biosynthesis, metabolism and role in living organisms. Crit. Rev. Food Sci. Nutr..

[32] dos Santos Rocha C., Magnani M., Jensen Klososki S., Aparecida Marcolino V., dos Santos Lima M., Queiroz de Freitas M., Carla Feihrmann A., Eduardo Barão C., Colombo Pimentel T. (2023). High-intensity ultrasound influences the probiotic fermentation of Baru almond beverages and impacts the bioaccessibility of phenolics and fatty acids, sensory properties, and in vitro biological activity. Food Res. Int..

[33] Wan X., Wu J., Wang X., Cui L., Xiao Q. (2024). Accumulation patterns of flavonoids and phenolic acids in different colored sweet potato flesh revealed based on untargeted metabolomics. Food Chem. X.

[34] Pang L.L., Chen L., Jiang Y.Q., Zhou C., Liang F.H., Duan L.H. (2023). Role of exogenous melatonin in quality maintenance of sweet cherry: Elaboration in links between phenolic and amino acid metabolism. Food Biosci..

[35] Clifford M.N., Ludwig I.A., Pereira-Caro G., Zeraik L., Borges G., Almutairi T.M., Dobani S., Bresciani L., Mena P., Gill C.I.R. (2024). Exploring and disentangling the production of potentially bioactive phenolic catabolites from dietary (poly)phenols, phenylalanine, tyrosine and catecholamines. Redox Biol..

[36] Portu J., López-Alfaro I., Gómez-Alonso S., López R., Garde-Cerdán T. (2015). Changes on grape phenolic composition induced by grapevine foliar applications of phenylalanine and urea. Food Chem..

[37] Chen Y., Pan H., Hao S., Pan D., Wang G., Yu W. (2021). Evaluation of phenolic composition and antioxidant properties of different varieties of Chinese citrus. Food Chem..

[38] Azuma A., Yakushiji H., Koshita Y., Kobayashi S. (2012). Flavonoid biosynthesis-related genes in grape skin are differentially regulated by temperature and light conditions. Planta.

[39] Androutsopoulos V.P., Papakyriakou A., Vourloumis D., Tsatsakis A.M., Spandidos D.A. (2010). Dietary flavonoids in cancer therapy and prevention: Substrates and inhibitors of cytochrome P450 CYP1 enzymes. Pharmacol. Ther..

[40] Zhu C., Zhou X., Long C., Du Y., Li J., Yue J., Pan S. (2020). Variations of flavonoid composition and antioxidant properties among different cultivars, fruit tissues and developmental stages of Citrus fruits. Chem. Biodivers..

[41] Carr A.C., Rosengrave P.C., Bayer S., Chambers S., Mehrtens J., Shaw G.M. (2017). Hypovitaminosis C and vitamin C deficiency in critically ill patients despite recommended enteral and parenteral intakes. Crit. Care.

[42] Maiani G., Periago Castón M.J., Catasta G., Toti E., Cambrodón I.G., Bysted A., Granado-Lorencio F., Olmedilla-Alonso B., Knuthsen P., Valoti M. (2009). Carotenoids: Actual knowledge on food sources, intakes, stability and bioavailability and their protective role in humans. Mol. Nutr. Food Res..

[43] Bernstein P.S., Li B., Vachali P.P., Gorusupudi A., Shyam R., Henriksen B.S., Nolan J.M. (2016). Lutein, zeaxanthin, and meso-zeaxanthin: The basic and clinical science underlying carotenoid-based nutritional interventions against ocular disease. Prog. Retin. Eye Res..

[44] Assefa A.D., Saini R.K., Keum Y.-S. (2017). Fatty acids, tocopherols, phenolic and antioxidant properties of six citrus fruit species: A comparative study. J. Food Meas. Charact..

[45] Matsuo Y., Miura L.A., Araki T., Yoshie-Stark Y. (2019). Proximate composition and profiles of free amino acids, fatty acids, minerals and aroma compounds in Citrus natsudaidai peel. Food Chem..

[46] Kasiotis K.M., Baira E., Iosifidou S., Manea-Karga E., Tsipi D., Gounari S., Theologidis I., Barmpouni T., Danieli P.P., Lazzari F. (2023). Fingerprinting chemical markers in the mediterranean orange Blossom Honey: UHPLC-HRMS metabolomics study integrating melissopalynological analysis, GC-MS and HPLC-PDA-ESI/MS. Molecules.

[47] Drewnowski A., Smith J., Fulgoni V.L. (2021). The new hybrid nutrient density score NRFh 4:3:3 tested in relation to affordable nutrient density and healthy eating index 2015: Analyses of NHANES data 2013-16. Nutrients.

[48] Fu H., Lee C.H., Nolden A.A., Kinchla A.J., Chen B. (2024). Nutrient density, added sugar, and fiber content of commercially available fruit snacks in the United States from 2017 to 2022. Nutrients.

[49] Drewnowski A., Fulgoni V.L. (2020). New nutrient rich food nutrient density models that include nutrients and myplate food groups. Front. Nutr..

[50] Lin J.J., Hui D.F., Kumar A., Yu Z.G., Huang Y.H. (2023). Climate change and/or pollution on the carbon cycle in terrestrial ecosystems. Front. Environ. Sci..

