# Supplementary material for: Untargeted Metabolomics Comparison and Nutrition Evaluation of Geographical Indication Newhall Navel Oranges in China
Source: Foods. 2025 Jan 22;14(3):355. doi: 10.3390/foods14030355 (PMC11816377; doi:10.3390/foods14030355)
Supplement: Supplementary file 1 [file foods-14-00355-s001.zip › foods-3397659-supplementary.pdf]

Table S1 The details of 114 detected compounds with VIP>1 and *p*<0.01 in the peeled citrus group

No.	name	ontology	rt(min)	*m/z*	formula	mass error（ppm）	*P*.value	VIP	mode
1	2-Isopropylmalic acid	organic acid	3.13	175.0594	C7H12O5	10.28	0.000460821	2.68	neg
2	Succinic acid		1.60	117.017	C4H6O4	8.56	0.000393669	2.64	neg
3	6-Hydroxyhexanoic acid		5.62	133.0864	C6H12O3	3.60	3.57128E-05	2.61	pos
4	Citric acid		1.34	191.0195	C6H8O7	1.17	0.000453961	2.43	neg
5	(S)-Methylmalonic acid semialdehyde		4.09	102.0345	C4H6O3	0.09	0.000489317	2.35	pos
6	4-Pyridoxic acid		0.56	182.9847	C8H9NO4	3.60	0.001553104	2.11	pos
7	Maleic acid		15.39	115.0014	C4H4O4	7.40	0.001455363	2.09	neg
8	6-Phosphogluconic acid		12.56	257.2115	C6H13O10P	1.78	0.001203526	1.93	neg
9	Geniposidic acid		7.72	355.1009	C16H22O10	5.66	0.002554241	1.89	neg
10	1,7-Dimethyluric acid		0.84	196.0597	C7H8N4O3	0.31	0.013266675	1.68	neg
11	4,5-Dihydroorotic acid		15.75	158.9617	C5H6N2O4	3.07	0.016739234	1.66	pos
12	(S)-Abscisic acid		5.37	265.1446	C15H20O4	4.24	0.002192967	1.58	pos
13	Methyl jasmonate		15.92	223.9872	C13H20O3	2.24	0.040660426	1.44	pos
14	Perillic acid		6.12	167.1079	C10H14O2	6.62	0.039656516	1.43	pos
15	Porphobilinogen		12.67	226.1807	C10H14N2O4	0.63	0.012778388	1.35	pos
16	3-Hydroxymethylglutaric acid		15.38	144.9826	C6H10O5	1.86	0.028940884	1.31	pos
17	Methyloxaloacetate		2.84	147.0291	C5H6O5	1.16	0.006036083	1.30	pos
18	L-threo-3-Methylmalate		14.96	129.0173	C5H8O5	11.70	0.035172175	1.24	neg
19	5-Hydroxyindoleacetic acid		15.21	191.054	C10H9NO3	21.98	0.001028144	1.13	neg
20	Quinate		1.46	191.0528	C7H12O6	13.17	0.02490965	1.12	neg
21	Terephthalate		3.43	165.0171	C8H6O4	13.48	0.048195	1.08	neg
22	Arachidic acid	fatty acid	12.76	311.1673	C20H40O2	3.52	0.025926232	1.56	neg
23	Stearidonic acid		13.46	277.2163	C18H28O2	0.36	0.03413268	1.34	pos
24	L-Aspartic acid	amino acid	1.51	134.0452	C4H7NO4	0.68	3.57128E-05	2.88	pos
25	L-Glutamic gamma-semialdehyde		5.54	131.0495	C5H9NO3	16.77	0.000485661	2.51	pos
26	D-beta-Phenylalanine		15.51	164.0701	C9H11NO2	9.90	0.000205513	2.49	neg
27	N-Succinyl-2-amino-6-ketopimelate		3.60	289.092	C11H15NO8	5.76	0.001455363	2.00	pos
28	N(6)-Methyllysine		6.39	161.1326	C7H16N2O2	0.14	0.002192967	1.85	pos
29	Glycylleucine		4.38	189.124	C8H16N2O3	3.30	0.001144829	1.82	pos

30	Diaminopimelic acid		4.36	190.1077	C7H14N2O4	0.14	0.007390943	1.75	pos
31	(S)-5-Amino-3-oxohexanoate		1.61	146.0816	C6H11NO3	1.72	0.000461975	1.68	pos
32	D-Asparagine		1.53	132.0479	C4H8N2O3	4.77	0.014958135	1.67	neg
33	2-Methylserine		2.49	119.0497	C4H9NO3	0.01	0.000802724	1.66	pos
34	L-Serine		1.49	106.0506	C3H7NO3	2.10	0.002547864	1.65	pos
35	L-4-Hydroxyphenylglycine		4.39	168.066	C8H9NO3	2.39	0.032386941	1.61	pos
36	gamma-Glutamylcysteine		13.80	250.1435	C8H14N2O5S	4.07	0.006425377	1.49	neg
37	Ergothioneine		2.98	231.098	C9H16N3O2S	24.13	0.005558094	1.47	pos
38	S-Adenosylmethionine		1.48	399.144	C15H22N6O5S	0.36	0.049291679	1.43	pos
39	N-Acetylleucine		2.94	172.0963	C8H15NO3	1.21	0.013467175	1.42	neg
40	N2-Malonyl-D-tryptophan		5.15	271.0692	C14H14N2O5	10.00	0.010280566	1.37	neg
41	3-Sulfinoalanine		15.67	153.0113	C3H7NO4S	11.11	0.040660426	1.31	pos
42	L-Methionine		2.19	150.058	C5H11NO2S	1.84	0.007065723	1.18	pos
43	DL-Glutamate		1.83	146.0442	C5H9NO4	11.80	0.040660426	1.13	neg
44	L-Tyrosine		3.45	182.0819	C9H11NO3	1.79	0.036607578	1.08	pos
45	Amygdalin		5.65	438.1313	C20H27NO11	19.88	0.000187356	2.39	neg
46	6-Deoxy-L-galactose		0.87	164.0641	C6H12O5	26.82	0.000511839	2.36	pos
47	D-Arabitol		3.88	153.0761	C5H12O5	1.67	0.000451697	2.34	pos
48	2-Deoxystreptamine		8.77	163.1121	C6H14N2O3	0.36	0.000433985	2.32	pos
49	N-Acetyl-D-glucosamine		3.00	204.0857	C8H15NO6	15.85	0.002766977	2.00	pos
50	Ribose 1,5-bisphosphate		12.05	309.1715	C5H12O11P2	8.52	0.001159229	1.98	neg
51	Raffinose		2.21	503.1578	C18H32O16	7.80	0.017120135	1.87	neg
52	Cellobiose	sugar	2.41	323.0967	C12H22O11	3.44	0.010753758	1.67	neg
53	D-Mannose		1.52	181.071	C6H12O6	1.79	0.035172175	1.59	pos
54	D-Maltose		3.91	342.1403	C12H22O11	2.95	0.028510011	1.60	pos
55	D-Ribose 5-phosphate		11.35	230.2481	C5H11O8P	1.56	0.000961035	1.55	pos
56	trans-Zeatin-7-beta-D-glucoside		4.81	382.1711	C16H23N5O6	2.55	0.010753758	1.53	pos
57	4-O-beta-D-Glucosyl-4-hydroxycinnamate		4.70	327.108	C15H18O8	1.60	0.001243283	1.34	pos
58	L-Rhamnono-1,4-lactone		2.39	163.0616	C6H10O5	8.08	0.029896914	1.04	pos
59	L-Fucose		1.26	164.0688	C6H12O5	1.83	0.046421155	1.04	pos
60	Hesperetin		10.27	301.0707	C16H14O6	2.28	0.000451697	2.59	neg
61	Orientin	flavonoid	6.51	447.0902	C21H20O11	6.90	0.004482833	2.27	neg
62	Poncirin		8.80	593.183	C28H34O14	7.72	0.002829939	2.09	neg

63	Isovitexin 2"-O-beta-D-glucoside		6.37	595.1653	C27H30O15	0.80	0.003543997	2.02	pos
64	Eriocitrin		6.89	595.1648	C27H32O15	3.40	0.00628242	1.97	neg
65	Vitexin		6.89	431.0942	C21H20O10	9.52	0.00162459	1.86	neg
66	Luteoforol		4.15	291.0851	C15H14O6	4.04	4.86918E-05	1.52	pos
67	(S)-Pinocembrin		8.72	257.0808	C15H12O4	0.14	0.022314915	1.39	pos
68	Quercetin 3-O-glucoside		7.04	463.0851	C21H20O12	6.75	0.005642094	1.22	neg
69	Hesperetin 7-neohesperidoside		6.54	611.2066	C28H34O15	15.58	0.019496896	1.14	pos
70	Hesperetin 7-O-glucoside		7.64	463.1214	C22H24O11	6.96	0.001051539	1.02	neg
71	Rutin		6.88	611.168	C27H30O16	11.98	0.003735028	1.01	pos
72	Hydrocinnamic acid	phenolic acid	6.19	133.0646	C9H10O2	29.56	3.57128E-05	2.55	pos
73	4-Hydroxycinnamic acid		14.15	146.9807	C9H8O3	4.69	0.000393669	2.46	pos
74	trans-2-Hydroxycinnamate		5.49	165.0542	C9H8O3	2.28	0.000461975	2.01	pos
75	Gallic acid		7.40	153.0186	C7H6O5	29.63	0.000408712	1.91	pos
76	trans-Cinnamate		12.90	131.0496	C9H8O2	2.40	0.001440882	1.77	pos
77	Dehydroascorbate	vitamin	1.99	173.0067	C6H6O6	6.52	0.000741007	2.49	neg
78	Dethiobiotin		15.92	213.9831	C10H18N2O3	2.67	0.005353525	1.42	pos
79	beta-Carotene		14.81	536.1655	C40H56	0.85	0.025540241	1.30	pos
80	4-Aminocatechol	Phenolic	16.43	126.0553	C6H7NO2	1.75	0.000322309	2.13	pos
81	4-Hydroxy-3-(3-methyl-2-butenyl)acetophenone		5.60	205.1226	C13H16O2	1.43	0.001440882	2.12	pos
82	Norepinephrine		15.69	169.9777	C8H11NO3	0.04	0.002787807	1.85	pos
83	2-Naphthol		15.50	144.0659	C10H8O	1.93	0.016739234	1.54	pos
84	Phenylephrine		2.49	150.0909	C9H13NO2	24.88	0.018499714	1.53	pos
85	Homovanillin		16.36	167.0708	C9H10O3	3.14	0.022092878	1.53	pos
86	O-Phosphoethanolamine	ester compound	1.31	141.9582	C2H8NO4P	3.38	0.002226109	1.74	pos
87	Neocnidilide		10.39	195.1386	C12H18O2	1.90	0.031194921	1.67	pos
88	(+)-Bornyl diphosphate		5.62	295.0448	C10H20O7P2	17.66	0.008804432	1.29	neg
89	Confertifolin		8.03	235.17	C15H22O2	1.15	0.038871265	1.18	pos
90	Alantolactone		9.70	233.1543	C15H20O2	2.20	0.000296786	1.18	pos
91	Nifedipine		12.67	329.1018	C17H18N2O6	22.08	0.003312673	1.17	pos
92	Limonin	limonoid	11.43	471.2006	C26H30O8	1.49	0.01091628	1.10	pos
93	1,2-Dehydroreticuline	alkaloid	4.20	328.1611	C19H22NO4	18.89	0.004416093	1.78	pos
94	5-Hydroxymethylfurfuryl alcohol	alcohol	2.96	128.0435	C6H8O3	29.68	0.01091628	1.89	pos
95	Pantetheine		5.82	277.1208	C11H22N2O4S	6.94	0.040864237	1.67	neg

96	Nandrolone		13.11	275.2012	C18H26O2	2.27	0.004783896	1.49	pos
97	Capsidiol		8.20	219.1749	C15H24O2	0.00	0.01545226	1.08	pos
98	6-Thioguanosine monophosphate	nucleoside	5.67	379.0631	C10H14N5O7PS	1.15	0.002065258	1.97	pos
99	Uridine		3.75	243.0614	C9H12N2O6	2.20	0.010753758	1.85	neg
100	Dehypoxanthine futalosine		1.50	296.0653	C14H16O7	1.40	0.011333413	1.69	pos
101	Guanosine		4.13	282.0834	C10H13N5O5	2.51	0.002192967	1.43	neg
102	N-D-Ribosylpurine		1.55	251.0764	C10H12N4O4	8.86	0.01545226	1.41	neg
103	Adenosine		3.99	268.1047	C10H13N5O4	2.33	0.013266675	1.14	pos
104	(-)-alpha-Curcumene	Terpene	10.72	203.1803	C15H22	2.18	0.001258921	1.44	pos
105	(1S,4R)-1-Hydroxy-2-oxolimonene		11.23	151.1118	C10H16O2	28.02	0.012494084	1.42	pos
106	Dihydrouracil	other	1.61	115.0393	C4H6N2O2	0.01	0.000461975	2.18	pos
107	Acetylphosphate		11.59	139.9881	C2H5O5P	1.40	0.005130768	2.03	pos
108	1-Naphthylamine		0.86	123.9004	C10H9N	4.42	0.028085552	1.69	neg
109	Aspulvinone E		1.54	295.0657	C17H12O5	15.17	0.005475345	1.63	neg
110	cis-Zeatin riboside		7.17	351.1647	C15H21N5O5	29.62	0.038484489	1.27	neg
111	trans-Zeatin riboside		5.92	351.164	C15H21N5O5	27.62	0.022148179	1.24	neg
112	Ubiquinone-1		6.58	233.1179	C14H18O4	20.70	0.000205513	1.15	pos
113	Zerumbone		6.98	219.1749	C15H22O	2.39	0.014516055	1.13	pos
114	Nootkatone		13.37	219.1749	C15H22O	2.39	0.0057417	1.07	pos

Table S2 The details of 85 detected compounds with VIP>1 and *p*<0.01 in the whole citrus group

**No.**	**name**	**ontology**	**rt(min)**	***m/z***	**formula**	**mass error（ppm）**	***P*.value**	**VIP**	**mode**
1	2-Isopropylmalic acid	organic acid	3.13	175.0594	C7H12O5	10.28	0.00117088	2.21	neg
2	Terephthalate		3.43	165.0171	C8H6O4	13.48	0.009279014	1.76	neg
3	2-Caffeoylisocitrate		4.71	353.0499	C15H14O10	4.32	0.021333002	1.45	neg
4	Methyloxaloacetate		2.84	147.0291	C5H6O5	1.16	0.049662756	1.60	pos
5	Maleic acid		15.39	115.0014	C4H4O4	7.40	0.040864237	1.56	neg
6	4-Hydroxycinnamic acid		14.15	146.9807	C9H8O3	4.69	0.000520875	1.43	pos
7	Porphobilinogen		12.67	226.1807	C10H14N2O4	0.63	0.000637791	1.37	pos
8	p-Anisic acid		16.41	153.055	C8H8O3	0.49	0.000341385	1.29	pos
9	Azelaic acid		15.66	170.9805	C9H16O4	25.52	0.000243596	1.15	pos

10	Glutaric acid		1.02	131.9747	C5H8O4	3.92	0.000302781	1.12	pos
11	Eprosartan		5.00	424.1423	C23H24N2O4S	8.02	0.001316867	1.09	neg
12	3-Hydroxymethylglutaric acid		15.38	144.9826	C6H10O5	1.86	0.037160831	1.07	pos
13	13-L-Hydroperoxylinoleic acid		13.10	293.2112	C18H32O4	1.74	0.003422103	1.60	neg
14	16-Hydroxy hexadecanoic acid	fatty acid	12.75	271.2266	C16H32O3	4.51	0.00761603	1.54	neg
15	9(S)-HPOT		12.74	293.2116	C18H30O4	0.86	0.023459025	1.49	pos
16	Alpha-dimorphecolic acid		13.64	295.2267	C18H32O3	3.81	0.000790773	1.07	neg
17	L-Glutamic gamma-semialdehyde		5.54	131.0495	C5H9NO3	16.77	0.003271522	2.17	pos
18	L-Aspartic acid		1.51	134.0452	C4H7NO4	0.68	0.000243596	2.04	pos
19	N-Acetylleucine		2.94	172.0963	C8H15NO3	1.21	0.002149543	1.73	neg
20	Asymmetric dimethylarginine		1.65	203.1525	C8H18N4O2	10.83	0.006621059	1.69	pos
21	Ketoleucine		15.43	131.534	C6H10O3	3.62	0.013003978	1.58	pos
22	Procollagen 5-hydroxy-L-lysine		8.58	197.8064	C7H13N3O3R2	8.31	0.021763958	1.56	neg
23	L-Alanyl-gamma-D-glutamyl-L-lysine	amino acid	4.94	346.1953	C14H26N4O6	3.48	0.008716827	1.32	pos
24	Creatine		1.03	130.9669	C4H9N3O2	1.81	0.046537353	1.24	pos
25	(S)-5-Amino-3-oxohexanoate		1.61	146.0816	C6H11NO3	1.72	0.002454088	1.19	pos
26	N(6)-Methyllysine		6.39	161.1326	C7H16N2O2	0.14	0.01545226	1.19	pos
27	Aspartylglycosamine		7.85	335.1327	C12H21N3O8	0.60	0.00148848	1.05	neg
28	Diaminopimelic acid		4.36	190.1077	C7H14N2O4	0.14	0.001159229	1.02	pos
29	beta-N-Acetylglucosamine		12.08	204.0857	C8H15NO6	15.85	0.001194533	1.78	pos
30	Trehalose		1.71	342.1113	C12H22O11	1.41	0.041481821	1.67	neg
31	D-Lyxose		8.50	149.0435	C5H10O5	4.63	0.001665716	1.67	neg
32	D-Ribose 5-phosphate		11.35	230.2481	C5H11O8P	1.56	0.031039335	1.64	pos
33	2'-N-Acetylparomamine		7.98	366.175	C14H27N3O8	1.34	0.014735437	1.57	pos
34	1-Kestose		2.38	503.1576	C18H32O16	8.20	0.010753758	1.56	neg
35	L-Fucose	sugar	1.26	164.0688	C6H12O5	1.83	0.00370712	1.52	pos
36	6-Deoxy-L-galactose		0.87	164.0641	C6H12O5	26.82	0.001356971	1.47	pos
37	Maltotriose		5.49	485.1627	C18H32O16	24.92	0.018499714	1.47	neg
38	Sorbitol		0.86	182.9865	C6H14O6	0.39	0.005784924	1.37	pos
39	1-O-Sinapoyl-beta-D-glucose		3.10	385.1088	C17H22O10	13.56	0.009209682	1.33	neg
40	L-Fucono-1,5-lactone		9.27	145.0484	C6H10O5	20.92	0.017034748	1.26	pos
41	Ribose 1,5-bisphosphate		12.05	309.1715	C5H12O11P2	8.52	0.010780676	1.24	neg

42	D-Maltose		3.91	342.1403	$C_{12}H_{22}O_{11}$	2.95	0.046537353	1.22	pos
43	Raffinose		2.21	503.1578	$C_{18}H_{32}O_{16}$	7.80	0.006081524	1.19	neg
44	Sucrose		3.55	341.1015	$C_{12}H_{22}O_{11}$	21.69	0.012778388	1.15	neg
45	trans-Zeatin-7-beta-D-glucoside		4.81	382.1711	$C_{16}H_{23}N_5O_6$	2.55	0.035793105	1.12	pos
46	L-Rhamnono-1,4-lactone		2.39	163.0616	$C_6H_{10}O_5$	8.08	0.00022656	1.06	pos
47	Abscisic acid glucose ester		5.37	427.1958	$C_{21}H_{30}O_9$	1.11	0.000451697	1.04	pos
48	Isovitexin 2''-O-beta-D-glucoside	flavonoid	6.37	595.1653	$C_{27}H_{30}O_{15}$	0.80	0.000336302	2.77	pos
49	Isovitexin		7.03	433.1109	$C_{21}H_{20}O_{10}$	4.56	0.000472487	2.51	pos
50	Diosmetin		10.26	301.071	$C_{16}H_{12}O_6$	1.08	0.000433985	1.98	pos
51	Nobiletin		11.66	403.1393	$C_{21}H_{22}O_8$	1.30	0.006081524	1.60	pos
52	Poncirin		8.80	593.183	$C_{28}H_{34}O_{14}$	7.72	0.027119561	1.35	neg
53	Hesperetin 7-O-glucoside		7.64	463.1214	$C_{22}H_{24}O_{11}$	6.96	0.000433985	1.32	neg
54	Aromadendrin		6.88	289.0711	$C_{15}H_{12}O_6$	1.47	0.01777433	1.29	pos
55	Catechin		1.53	290.0761	$C_{15}H_{14}O_6$	10.00	0.001782034	1.23	pos
56	Kaempferol-3-O-rutinoside		7.28	595.1718	$C_{27}H_{30}O_{15}$	10.12	0.024173459	1.09	pos
62	trans-2-Hydroxycinnamate	phenolic acid	5.49	165.0542	$C_9H_8O_3$	2.28	0.00162459	2.22	pos
63	trans-Cinnamate		12.90	131.0496	$C_9H_8O_2$	2.40	0.003439256	2.18	pos
57	Hydrocinnamic acid		6.19	133.0646	$C_9H_{10}O_2$	29.56	0.000243596	1.78	pos
58	Sinapic acid		4.95	223.0602	$C_{11}H_{12}O_5$	4.48	0.013003978	1.51	neg
59	Gallic acid		7.40	153.0186	$C_7H_6O_5$	29.63	0.000585819	1.34	pos
60	Gentisic acid		15.46	154.9901	$C_7H_6O_4$	0.31	0.00012341	1.30	pos
61	trans-Ferulic acid		11.81	195.0652	$C_{10}H_{10}O_4$	0.06	0.010435936	1.18	pos
64	beta-Carotene	vitamin	14.81	536.1655	$C_{40}H_{56}$	0.85	0.000326363	1.85	pos
65	Ascorbate		1.51	176.0264	$C_6H_8O_6$	4.41	0.02547647	1.65	neg
66	Dehydroascorbate		1.99	173.0067	$C_6H_6O_6$	6.52	0.000802724	1.44	neg
67	Nicotinamide riboside		2.14	256.1033	$C_{11}H_{15}N_2O_5$	8.11	0.025540241	1.38	pos
68	4-Aminocatechol	Phenolic	16.43	126.0553	$C_6H_7NO_2$	1.75	0.000460821	2.58	pos
69	Catechol		15.67	111.0443	$C_6H_6O_2$	2.02	0.026252344	1.44	pos
70	3-Hydroxybenzaldehyde		3.83	123.0447	$C_7H_6O_2$	3.10	0.036607578	1.08	pos
71	4-Hydroxybenzaldehyde		16.07	121.0272	$C_7H_6O_2$	19.20	0.00628242	1.04	neg
72	Dibutyl phthalate	ester compound	13.80	279.1595	$C_{16}H_{22}O_4$	1.49	3.57128E-05	1.33	pos
73	alpha-Santonin		9.71	247.1335	$C_{15}H_{18}O_3$	2.52	0.039656516	1.28	pos

74	(+)-Bornyl diphosphate		5.62	295.0448	C10H20O7P2	17.66	0.002192967	1.01	neg
75	Limonoate A-ring-lactone	limonoid	7.19	471.203	C26H32O9	12.38	0.013808099	1.42	pos
76	Vomilenine	alkaloid	5.97	349.148	C21H22N2O3	22.12	0.031039335	1.41	neg
77	Nornicotine		7.07	149.023	C9H12N2	2.12	0.000302781	1.36	pos
78	Deacetylisoipecoside		4.89	523.1985	C25H33NO11	13.19	0.001742386	1.02	neg
79	Sequoyitol	alcohol	5.00	194.0825	C7H14O6	5.25	0.010280566	1.45	pos
80	Vanylglycol		5.80	167.0706	C9H12O4	0.08	0.000294568	1.29	pos
81	Maltol		1.59	125.0221	C6H6O3	18.54	0.000349153	1.13	neg
82	Thymidine	nucleoside	11.53	223.0268	C10H14N2O5	4.90	0.030730489	1.54	neg
83	6-Thioguanosine monophosphate		5.67	379.0631	C10H14N5O7PS	1.15	0.001083564	1.08	pos
84	DHHA	other	4.97	156.0661	C7H9NO3	4.00	0.030500872	1.20	pos
85	Creatinine		12.96	112.9833	C4H7N3O	8.97	0.000388779	1.09	neg
